# Subthalamic 85 Hz deep brain stimulation improves walking pace and stride length in Parkinson’s disease patients

**DOI:** 10.1186/s42466-023-00263-7

**Published:** 2023-08-10

**Authors:** F. Mügge, U. Kleinholdermann, A. Heun, M. Ollenschläger, J. Hannink, D. J. Pedrosa

**Affiliations:** 1 Department of Neurology, University Hospital of Marburg, Baldingerstraße, Marburg, Germany; 2 Portabiles HealthCare Technologies, Henkestraße 91, 91052 Erlangen, Germany; 3 Center of Mind, Brain and Behaviour, Philipps University Marburg, Hans-Meerwein- Straße, Marburg, Germany

**Keywords:** Deep brain stimulation, Low frequency, Nucleus subthalamicus, Gait, Mobile sensors, Inertial measurement unit, Parkinson

## Abstract

**Background:**

Mobile gait sensors represent a compelling tool to objectify the severity of symptoms in patients with idiopathic Parkinson’s disease (iPD), but also to determine the therapeutic benefit of interventions. In particular, parameters of Deep Brain stimulation (DBS) with its short latency could be accurately assessed using sensor data. This study aimed at gaining insight into gait changes due to different DBS parameters in patients with subthalamic nucleus (STN) DBS.

**Methods:**

An analysis of various gait examinations was performed on 23 of the initially enrolled 27 iPD patients with chronic STN DBS. Stimulation settings were previously adjusted for either amplitude, frequency, or pulse width in a randomised order. A linear mixed effects model was used to analyse changes in gait speed, stride length, and maximum sensor lift.

**Results:**

The findings of our study indicate significant improvements in gait speed, stride length, and leg lift measurable with mobile gait sensors under different DBS parameter variations. Notably, we observed positive results at 85 Hz, which proved to be more effective than often applied higher frequencies and that these improvements were traceable across almost all conditions. While pulse widths did produce some improvements in leg lift, they were less well tolerated and had inconsistent effects on some of the gait parameters. Our research suggests that using lower frequencies of DBS may offer a more tolerable and effective approach to enhancing gait in individuals with iPD.

**Conclusions:**

Our results advocate for lower stimulation frequencies for patients who report gait difficulties, especially those who can adapt their DBS settings remotely. They also show that mobile gait sensors could be incorporated into clinical practice in the near future.

**Supplementary Information:**

The online version contains supplementary material available at 10.1186/s42466-023-00263-7.

## Background

Deep Brain Stimulation (DBS) of the subthalamic nucleus (STN) is an efficacious treatment for idiopathic Parkinson’s disease (iPD) [1]. In advanced cases with pronounced dyskinesias or tremor refractory to medical treatment [2], DBS may reduce symptom burden and promote long-term [3, 4] quality of life. The benefit of STN-DBS is well-documented, making it a feasible therapeutic option [5, 6]. Nevertheless, the benefit of DBS for symptoms beyond bradykinesia and tremor, especially gait, has been a topic of debate [7–9].

A major challenge for physicians treating iPD-patients remains the alleviation of axial motor symptoms as well as gait disorders [10]. With increasing disease duration, numerous patients develop walking difficulties with substantial repercussions on quality of life and frailty [11]. Treatments such as physical exercise and walking aids are indispensable to reduce the risk of falls with secondary complications [12]. Meanwhile, gait disturbances are rarely treated specifically. Even in initial DBS programming most centers prioritize upper limb tremor and rigidity relief over walking disturbances [13]. Reports about specific effects of tailored DBS to improve gait are either contradictory [14] or lack support by high quality data. Especially the claim of better control of axial symptoms via low-frequency stimulation [15–17] has not yet been substantially validated. A recent study did not report any differences in gait speed when testing 60 Hz vs. >100 Hz stimulation [18]. An approach to analyse the effect of short pulse width stimulation in comparison to conventional pulse width showed only equal efficiency [19]. The analysis of gait parameters however has received increasing appreciation recently. Nowadays, practically all smartphones or -watches can report gait information. Increasing miniaturisation with unobtrusive and durable sensors permitting continuous recordings has also opened many scientific possibilities [20, 21]. This applies particularly to individuals with iPD, who may eventually experience gait disturbances and develop fluctuations that necessitate personalized and adaptive therapies. In the future, the use of sensors could provide a reliable means of ensuring that such therapies are delivered effectively on a continuous basis saving time of physicians and caregivers. Therefore, we applied an already validated [22–24] system of mobile gait sensors to objectively measure gait changes in iPD-patients treated with chronic STN-DBS under different stimulation conditions.

## Patients and methods

The study was approved by the ethics committee of the University of Marburg (reference number 33/20) and conducted in accordance with the Declaration of Helsinki. All patients gave informed written consent before inclusion.

### Study Population

We included individuals between 18 and 75 years of age with a diagnosis of iPD based on clinical criteria [25] who had undergone DBS implantation with steerable electrodes in the STN at least three months prior to the study. Patients were recruited when visiting our clinic for a regular follow up, therefore no additional costs for travel or insurance were incurred. All patients had undergone a monopolar review previously as part of their routine follow-up to determine the most effective DBS stimulation parameters. We excluded minors, institutionalised persons, those unable to walk independently and individuals with more than two falls in the previous 30 days. Patients with neurological diseases such as epilepsy, Alzheimer’s disease or dystonia, or with severe psychiatric diseases, were also excluded. Finally, we excluded individuals with hearing or visual impairments that could significantly interfere with this study, and those pregnant or breastfeeding. In total, 27 patients participated in this study of which 23 (10 female) were subjected to the final analyses. Three subjects were excluded due to technical issues, whereas one participant discontinued participation because of unbearable impairments by DBS settings adjustments. Clinical details of the sample are shown in Table 1. During the study, patients were on regular medication. The majority (eighteen) of participants were chronically treated with a pulse frequency of 130 Hz, whereas three patients used a lower and two a higher frequency. The most common pulse width in chronic stimulation was 60 µs, while four subjects used a lower pulse width. Stimulation amplitudes ranged between 0.6 and 4.6 mA. Individual clinical details of each patient are available in supplementary Table 1.

**Table 1:** Clinical details of the patient population

	Overall
n	23
Age in years (mean (SD))	57 (7.27)
Gender = w (%)	10 (43.48)
Months post surgery (mean (SD))	17.04 (11.74)
UPDRS ON* (mean (SD))	14.95 (9.55)
UPDRS OFF* (mean (SD))	30.82 (14.98)
Amplitude left (mean (SD))	2.71 (0.99)
Amplitude right (mean (SD))	2.3 (0.89)
Frequency in Hz (%) left/right	
79	1 (4.3) / 1 (4.3)
100	1 (4.3) / 1 (4.3)
120	1 (4.3) / 1 (4.3)
130	18 (78.3) / 18 (78.3)
132	1 (4.3) / -
159	- / 1 (4.3)
185	1 (4.3) / 1 (4.3)
Impulse width in µs (%)	
30	1 (4.3)
40	1 (4.3)
50	2 (8.7)
60	19 (82.6)

Abbreviations: SD – standard deviation, UPDRS – Unified Parkinson’s Disease Rating Scale, Hz – Hertz, µs - Microseconds, * ON and OFF refers to stimulation switched on and off, while both conditions were measured with regular medication taken

### Study protocol

At the beginning of the experiment, patients had mobile gait sensors attached to both feet (cf. section gait assessment). DBS settings were then changed in a random order by altering one parameter (amplitude, frequency or pulse width) while leaving the others constant at the individuals’ chronic values (cf. supplementary Table 1). Nine settings, chosen to cover a wide range of different stimulation parameters, were tested: stimulation amplitude of 100%, 66% and at 33% of the original settings; stimulation frequencies of 30, 85 and 130 Hz and pulse widths of 40 and 90 µs. Additionally every patient was tested with DBS switched off (cf. supplementary Table 2 for an overview of the stimulation settings). Including more then nine conditions would have made the study too tiring for the participants. DBS changes were not disclosed to patients.

With each stimulation setting patients performed four gait exercises in a randomised order once: (i) normal walking, (ii) slow walking, (iii) fast walking (each 10 m in one direction, then turning around and walking back) as well as (iv) two minutes of free walking. Additionally, we conducted (v) a modified timed-up-and-go test (TUG) two times consecutively. In the TUG patients rise from a chair, walk for three metres, turn around 180°, walk back and sit down again. In our study walking distance was increased to ten metres. During all tests, sensor data was recorded continuously.

### Clinical assessment

Motor symptoms were assessed using part III of the revised version of the unified parkinson’s disease rating scale (MDS-UPDRS) [26] in two conditions: with no application of stimulation (OFF) and in the 100% amplitude condition (ON). Patients also rated which parameter setting was best and worst and reported side effects for all conditions. Patients could withdraw from a condition if the setting was intolerable. We could measure 83% of all settings but the rejected settings were not distributed evenly among conditions. For example, 14 out of 27 patients (52%) could not bear 90 µs pulse width. 30 Hz frequency and 33% of stimulation intensity were better tolerated, but still rejected by six and five out of 27 patients respectively (supplementary Table 3 gives an overview of not tolerated settings).

### Gait assessment

Gait data were acquired using the Mobile GaitLab home (Portabiles HealthCare Technologies GmbH, Version 1.2.0). The system features two inertial measurement units (IMUs) recording linear acceleration and angular rate data at 102.4 Hz and is approved as a class I medical device in germany for the purpose of measuring gait in Parkinson’s disease. An android device was used to wirelessly start and stop recordings and to transfer pseudonymized sensor data via Bluetooth. Time and order of DBS parameter settings and the gait task (slow, normal, fast and free walking; TUG) were noted manually. These annotations were used to split the recording into single gait tests using the python package MaD GUI with custom plugins [27].

Gait parameters were calculated by stride detection and subsequent parametrization. For the former we used dynamic time warping [28]. Stride parametrization entails gait event detection followed by a reconstruction of the foot’s orientation and trajectory [29]. From the detected gait events – mid-stance, toe-off and heel-strike – temporal gait parameters like the duration of stride, swing and stance phases are obtained. Then stride length, gait speed and maximum sensor lift as well as other gait parameters which were not subject of this study are calculated.

**Fig. 1:**
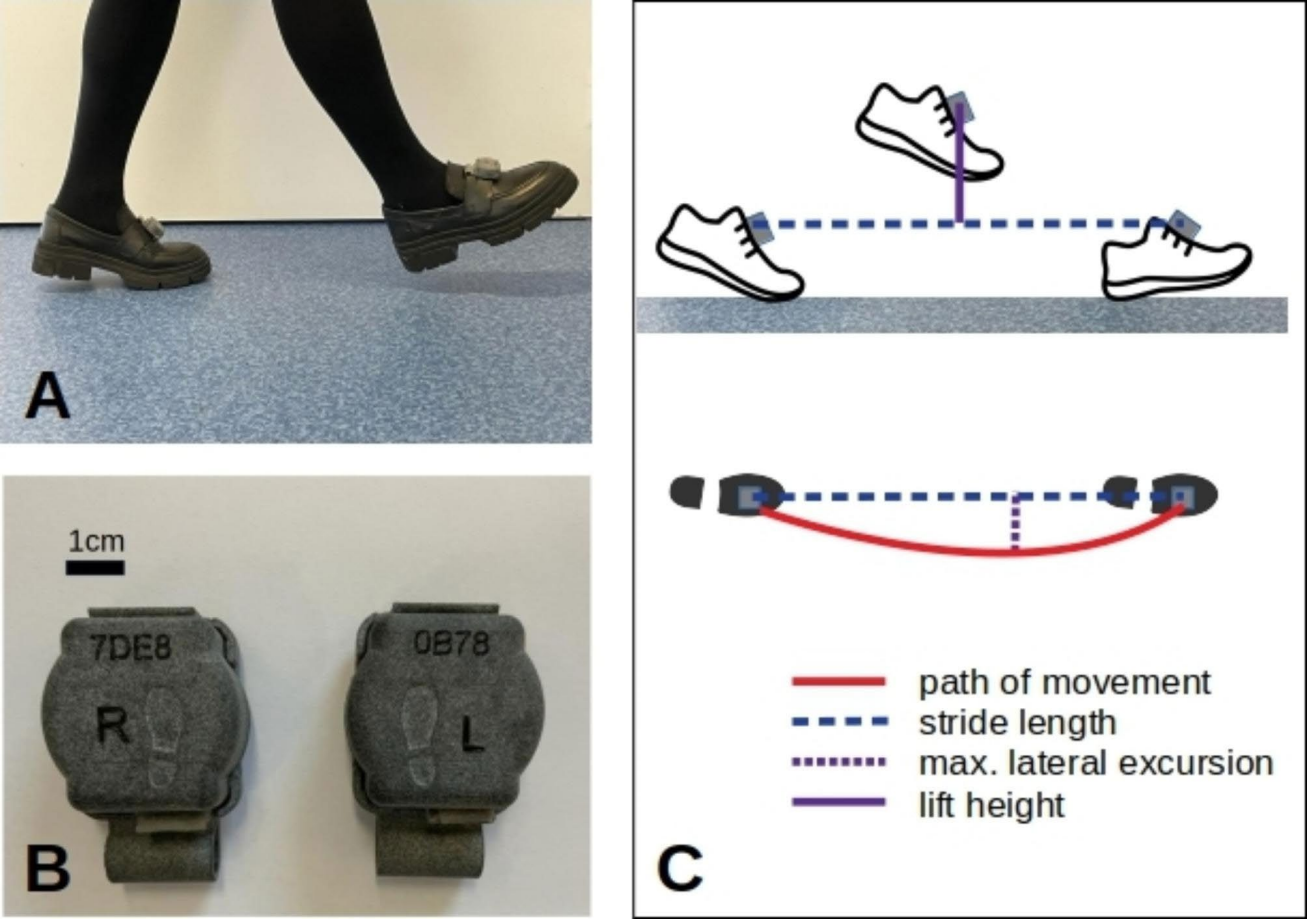
(**A**) Gait sensors as they were attached to the patient’s feet. (**B**) Close-up view of gait sensors with scale bar. (**C**) Schematic drawing of a step seen from the side and from the top which illustrates some of the gait parameters assessed by the gait sensor system

### Statistical analyses

Statistical analyses were conducted using R [30] (Version 3.6.3). First gross outliers, that is data three standard deviations above or below the mean, were removed. Since not all patients could be measured in all conditions and cell sizes were unequal due to different numbers of steps for each subject within a condition, we adopted linear mixed effects models [31] - implemented in the nlme package [32] (Version 3.1–144) - as an omnibus test instead of a repeated measures analysis of variance. We used the stimulation condition as the fixed effect of interest and the different gait conditions (normal, free, fast and slow) as well as the two timepoints of TUG measurement as covariates. Subject ID was used as a random factor. We allowed for heterogeneous variances between conditions because it resulted in a more homogeneous distribution of residuals. For post-hoc linear contrasts we applied the multcomp package [33] (Version 1.4–17) and corrected for multiple comparisons using a false discovery rate correction on a per analysis basis. All analyses and results can be found online in a GitHub repository (https://github.com/dpedrosac/DBSgait.)

## Results

For the clinical assessment, we report the MDS-UPDRS part III values as well as subjective ratings of patients on effectiveness and side effects of DBS settings. For the analysis of objective gait parameters we report gait speed, sensor lift and stride length. We chose these three parameters as specific and practical values with likely the highest practical importance for patients.

### Clinical assessments

#### MDS-UPDRS part III scores

All patients showed a better outcome in MDS-UPDRS part III with the stimulator switched on. The difference of 15.87 points between ON and OFF conditions was significant in a paired Wilcoxon signed rank test which we performed instead of a t-test due to inhomogeneous variances (V = 0, p < .001).

#### Subjective ratings

Ratings of DBS settings revealed a preference for the 130 Hz condition, the default setting of most participants (see supplementary Fig. 1 and supplementary Table 1). The 130 Hz condition for these participants was identical to the condition of 100% amplitude, which was the next setting in preference, followed by a pulse frequency of 85 Hz. Unsurprisingly most participants rejected the OFF condition, followed by the 90µs and 40µs pulse width conditions.

#### Side effects

Most frequently, subjects complained of feeling uneasy or unsettled (cf. Table 2). Another fairly common discomfort was fatigue. Fewer, but still a considerable number of participants, reported typical parkinsonian symptoms such as increased tremor, stiffness and speech difficulties. The remaining side effects were concentration problems, instability, paraesthesias, pain and muscle cramps.

**Table 2:** Reported side effects for each experimental condition. The numbers indicate the amount of participants reporting a particular side effect in the according condition. Rows are ordered by the summed occurrence across all conditions. Only side effects with a summed occurrence of at least ten were considered

	condition								
	amplitude (%)				frequency (Hz)			pulse width (µs)	
	OFF	33	66	100	30	85	130	40	90
uneasiness/insecurity	15	9	10	-	6	-	2	10	11
fatigue	4	7	5	-	10	-	-	5	6
tremor	9	3	3	-	4	1	1	5	1
rigidity	8	4	1	-	4	-	-	4	4
speech disturbance	2	3	-	1	3	1	1	3	5
difficulty concentrating	7	2	-	-	2	-	-	1	4
instability	4	2	1	-	3	-	2	3	-
paresthesia	2	2	1	-	-	1	-	2	4
pain	1	1	1	-	-	-	-	-	9
muscle cramps	-	-	3	1	-	-	-	2	4

### Gait assessment

**Fig. 2:**
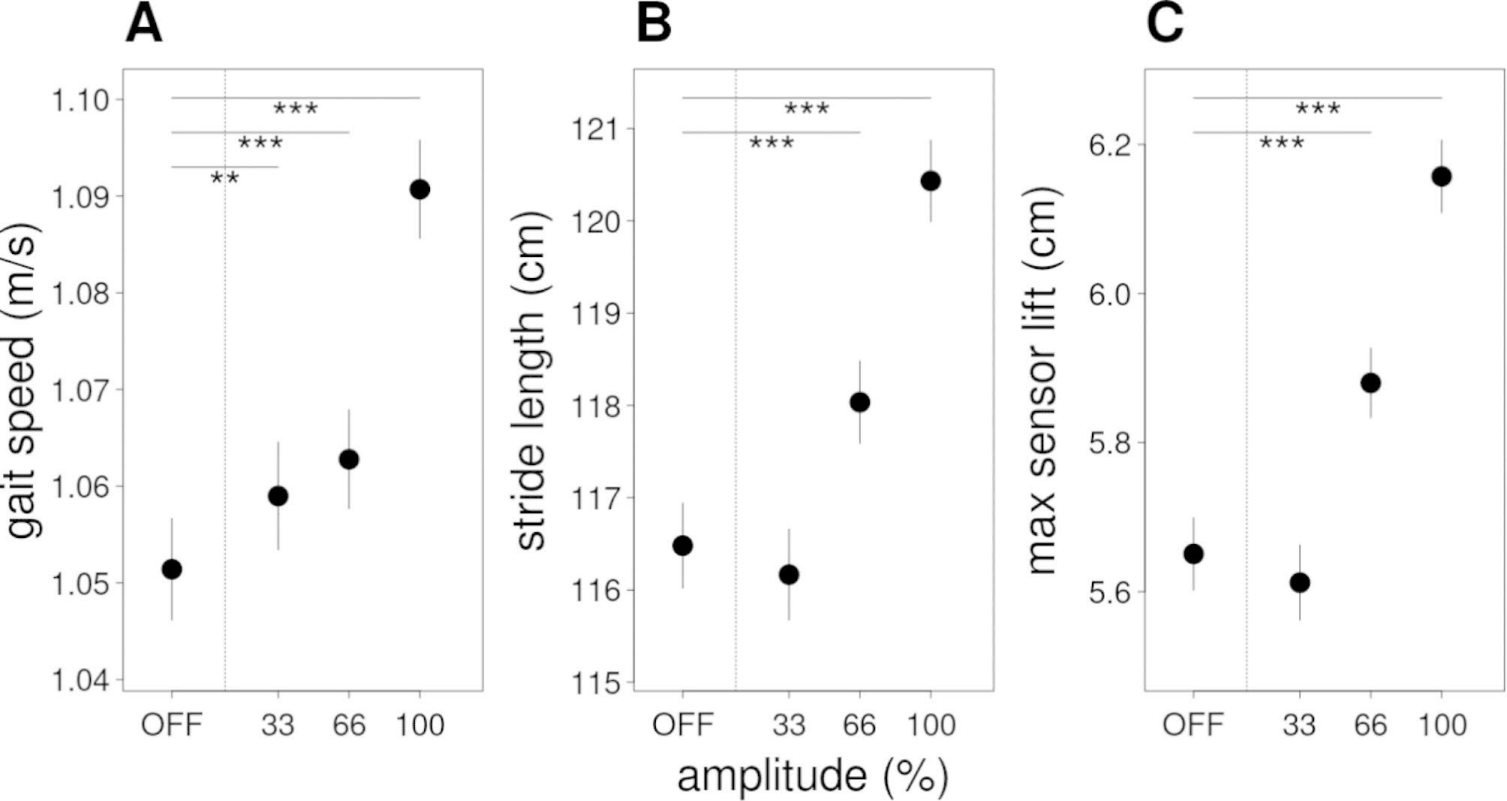
Estimates of gait parameters with different amplitude settings. The three panels display changes in gait speed (**A**), stride length (**B**) and maximum sensor lift (**C**) in dependence of stimulation amplitude. In the X-axis, DBS parameters are displayed at 33, 66 and 100% of the original patient’s amplitude, respectively. Error bars show two standard errors of the estimated difference between each condition and the reference condition (OFF). The error for the reference condition itself is estimated from the average of all other errors. Significant differences with respect to the OFF condition are indicated with * (p < .05), ** (p < .01) and *** (p < .001) respectively

To evaluate whether gait sensor data are in accordance with clinical findings, we assessed the different amplitude conditions in the pooled data of slow, fast, normal and free gait. In accordance with clinical measurements, sensor measurements at 100% amplitude surpassed the other settings and were especially better than in the “OFF” condition thus indicating good overall validity of the sensor recordings (cf. Figure 2). Gait speed estimates amounted to 1.051 m/s in the “OFF” condition and 1.091 m/s in the 100% condition, differing by Δ = 4 cm/s. For stride length and maximum sensor lift there was an almost linear improvement at increasing DBS amplitudes with differences of Δ = 3.952 cm and Δ = 0.506 cm between 100% and “OFF” respectively.

Consecutively, we analysed the different frequency and pulse width settings. The pooled data for slow, fast, normal and free gait and the TUG data were analysed separately. Please note that the TUG returned less data than the other conditions due to fewer steps needed to perform the test.

#### Gait speed all tasks

For gait speed of all tasks, except the TUG, there was an inverse U-shaped relationship for stimulation frequencies with 85 Hz leading to a significantly higher gait speed compared to 30 and to 130 Hz (Δ = 0.036 m/s, z = 13.441, p < .001 and Δ = 0.039 m/s, z = 15.771, p < .001, respectively). The latter two frequencies showed no significant difference (Δ = 0.003 m/s, z = 0.991, p = .382). This pattern of results was observed across all four tasks (cf. supplementary Fig. 2). Both tested pulse widths led to similar gait speed (Δ = 0.001 m/s, z = 0.366, p = .754) although this differed between conditions (cf. supplementary Fig. 2). 85 Hz stimulation showed not only the highest gait speed over all conditions but was significantly better than the baseline optimal DBS parameters (represented at 100% amplitude, Δ = 0.016 m/s, z = 6.614, p < .001). No significant difference between 33% and 66% of the original amplitude was detected (Δ = 0.004 m/s, z = 1.404, p = .203), while at 100% amplitude gait speed was significantly higher compared to the 66% condition (Δ = 0.028 m/s, z = 11.473, p < .001).

**Fig. 3:**
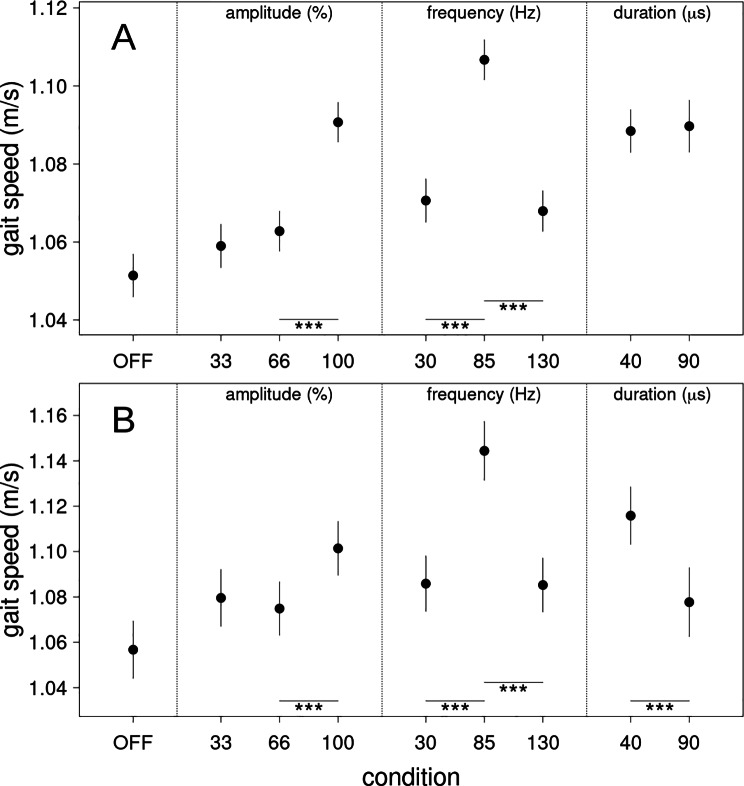
Gait speed estimates. (**A**) assessed from all gait tasks (slow, fast, normal and free gait) and (**B**) assessed from the timed-up-and-go test. Depicted are all measured stimulation conditions. OFF is no DBS stimulation, 33, 66 and 100 refer to the percentage in amplitude of the original stimulation settings of the patient. 30, 85 and 130 denote the stimulation frequency (Hz) whereas 40 and 90 refer to the stimulation pulse widths (µs). Error bars are two standard errors of the estimated difference between each condition and the reference condition (OFF). The error for the reference condition is estimated from the average of all other errors. Significant differences are indicated with * (p < .05), ** (p < .01) and *** (p < .001) respectively

#### Gait speed timed up and go test

In the TUG gait speed was also maximal at 85 Hz with 1.14 m/s. Analogous to the other gait tests (see above) gait speed at 85 Hz was faster than at 30 and 130 Hz (Δ = 0.059 m/s, z = 8.908, p < .001 and Δ = 0.059 m/s, z = 9.367, p < .001 respectively). 100% of amplitude led to higher gait speed in the TUG than stimulation with 66% (Δ = 0.027 m/s, z = 4.573, p < .001) but 66% differed not significantly from 33% stimulation amplitude (Δ = 0.005 m/s, z = 0.761, p = .471). Contrary to the other gait tests, in the TUG a pulse width of 40 µs significantly improved gait speed compared to 90 µs (Δ = 0.038 m/s, z = 4.89, p < .001) but only 13 patients tolerated 90 µs pulses.

**Fig. 4:**
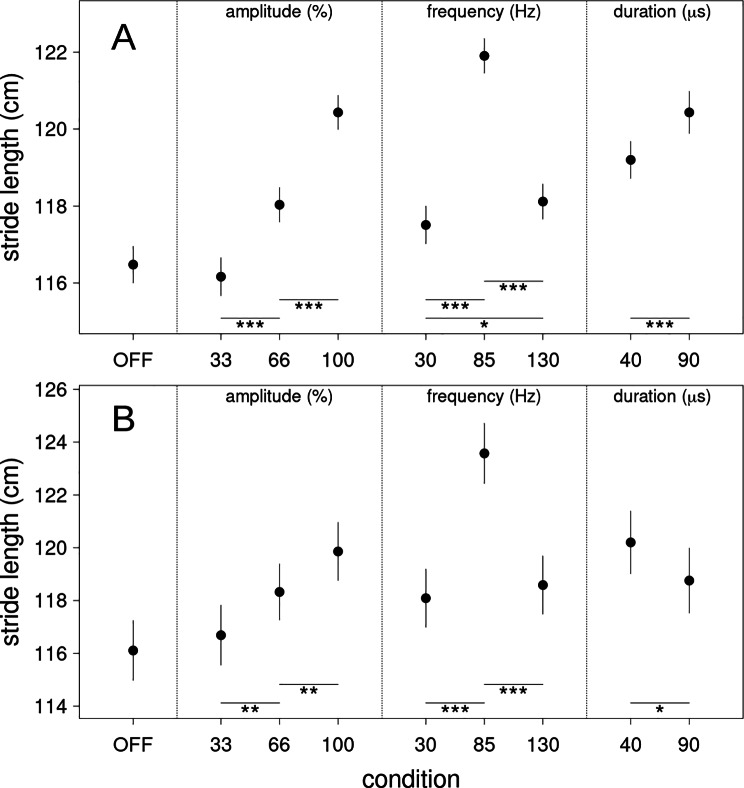
Stride length estimates. (**A**) assessed from all gait tasks (slow, fast, normal and free gait) and (**B**) assessed from the timed-up-and-go test. OFF is no DBS stimulation, 33, 66 and 100 refer to the percentage in amplitude of the original stimulation settings of the patient. 30, 85 and 130 denote the stimulation frequency (Hz) whereas 40 and 90 refer to the stimulation pulse widths (µs). Error bars are two standard errors of the estimated difference between each condition and the reference condition (OFF). The error for the reference condition is estimated from the average of all other errors. Significant differences are indicated with * (p < .05), ** (p < .01) and *** (p < .001) respectively

#### Stride length all tasks

Similar to gait speed, stride length across all gait tasks (slow, normal, fast free) also increased significantly at 85 Hz (Δ = 4.392 cm, z = 18.663, p < .001 and Δ = 3.785 cm, z = 17.658, p < .001 compared to 30 and 130 Hz respectively). Additionally, increasing amplitude linearly improved stride length. Contrary to gait speed, stride length was significantly higher at 90 µs pulse width compared to 40 µs (Δ = 1.233 cm, z = 4.394, p < .001).

#### Stride length timed up and go test

In the TUG, there also was a linear dependency of stride length to stimulation amplitude (cf. Figure 3). Contrary to the other gait tests stride length was longer at 40 µs compared to 90 µs (Δ = 1.445 cm, z = 2.205, p = .035).

#### Maximum sensor lift all tasks

**Fig. 5:**
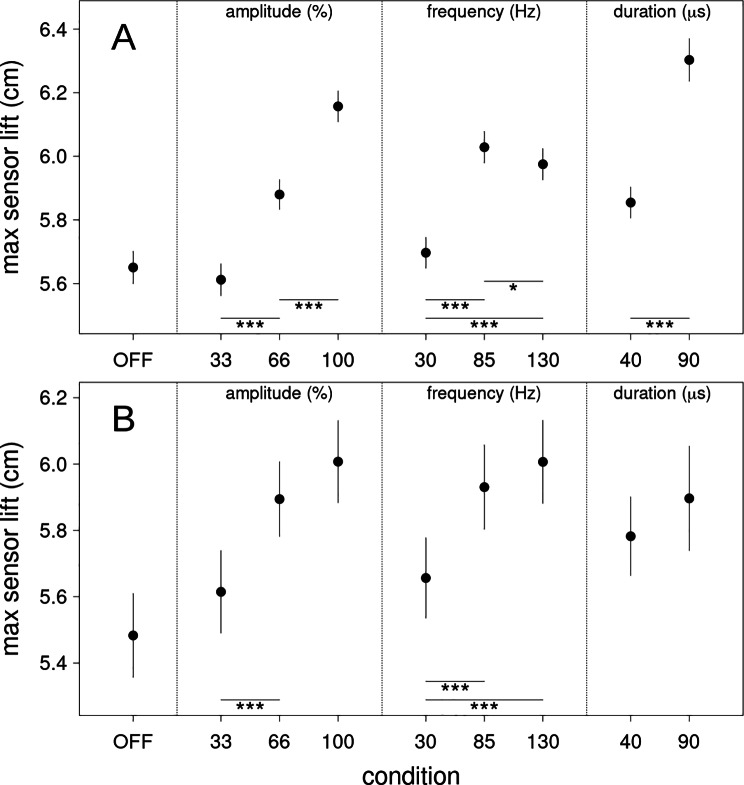
Maximum sensor lift estimates. (**A**) assessed from all gait tasks (slow, fast, normal and free gait) and (**B**) assessed from the timed-up-and-go test. OFF is no DBS stimulation, 33, 66 and 100 refer to the percentage in amplitude of the original stimulation settings of the patient. 30, 85 and 130 denote the stimulation frequency (Hz) whereas 40 and 90 refer to the stimulation pulse widths (µs). Error bars are two standard errors of the estimated difference between each condition and the reference condition (OFF). The error for the reference condition is estimated from the average of all other errors. Significant differences are indicated with * (p < .05), ** (p < .01) and *** (p < .001) respectively

The best condition with respect to maximum sensor lift in the gait tasks was a pulse width of 90 µs which even showed a significantly higher sensor lift than the predefined clinically best “ON” (denoted with “100% amplitude” in Fig. 5, Δ = 0.146 cm, z = 4.381, p < .001). With regard to frequency, 85 Hz stimulation improved sensor lift the most, showing significantly higher values than 30 Hz (Δ = 0.332 cm, z = 13.586, p < .001) and 130 Hz (Δ = 0.054 cm, z = 2.19, p = .032).

#### Maximum sensor lift timed up and go test

Analysis of maximum sensor lift during the TUG showed heterogeneous results. A nearly linear relationship between stimulation amplitude and maximum sensor lift was measured and no condition surpassed the setting of 100% stimulation amplitude. Nevertheless, pulse frequencies of 85 Hz led to higher sensor lift than those of 30 Hz (Δ = 0.274 cm, z = 4.296, p < .001) whereas 130 Hz was not inferior (Δ = 0.076 cm, z = 0.065, p = .253). Pulse widths did not differ in direct comparison (Δ = 0.114 cm, z = 1.474, p < .178).

## Discussion

We present a comprehensive analysis of the differential effects of DBS parameters on gait using mobile sensors. We provide compelling evidence that stimulation frequencies of 85 Hz - lower than the standard setting of 130 Hz - can significantly improve gait performance in iPD patients. These findings emphasise the potential of adapting stimulation frequencies to optimise therapeutic outcomes of neuromodulation in individuals suffering from iPD with gait disturbances.

Critical to relieving symptoms by DBS are precise lead localization and individually tailored current delivery. Nowadays, centres mostly rely on clinical examinations, imaging and, more recently, local field potential (LFP) recordings to adjust parameters [34, 35]. Axial symptoms and especially gait patterns are often not routinely considered. With respect to the aforementioned approaches this can be problematic considering the somatotopic organisation of target areas with the leg representation possibly at slightly distinct locations [36], but also because it remains unclear whether established markers such as beta activity correlate with gait disorders. Therefore, gait markers that indicate necessary treatment adjustments are needed.

Clinical data in general and sensor measurements specifically pose an easy to obtain feedback to close the loop in adaptive DBS systems [37]. The data presented here returned plausible clinical results as e.g. an improvement of objective gait measures with higher stimulation amplitude (cf. Figure 3). A big advantage is that such sensors are unobtrusive and easy to place and can record daily situations without major restrictions unlike LFP [38, 39] or electromyography data [40]. While designing our study, those aspects were taken into consideration using paradigms as close to everyday life as possible including a two minute free walking condition instead of relying solely on standardised walking tests. Our aim was to bridge the reported gap [41] between laboratory and real-world while maintaining a rigorous experimental design.

While our study demonstrated significant improvements in gait at lower DBS frequencies than most patients are using, the critical question is: are these improvements clinically relevant for patients’ walking ability? To answer this question, we compared the effect of changing the stimulation frequency from 130 Hz to 85 Hz with the gains in walking speed and stride length when increasing the amplitude from “OFF” to 100%. The improvement was similar in both scenarios. Furthermore a recent meta-analysis found a difference in walking speed between healthy individuals and iPD patients of 17 cm/s [42]. In our study the improvement in gait speed and stride length due to frequency reduction was about a quarter of this difference. Our results also suggest that frequency adjustment compensates for three years of disease progression in terms of walking speed, as 1.24 cm/s per year decrease in walking speed have been reported [43]. These comparisons support the clinical relevance and potential benefit of frequency tuning in DBS for patients with walking disability and underline the need to consider gait as a critical aspect of DBS therapy.

Our results provide valuable insights into the effects of DBS on gait performance in iPD-patients. However, some limitations apply: First, we cannot interpolate from our results possible side-effects or worsening of upper limb symptoms resulting from lower total energy delivered [44]. This is also a possible explanation why participants preferred the best clinical settings and raises the question if amplitude adjustments would be necessary to compensate for lower stimulation frequency. It is also possible that the effects of different DBS settings were masked by the fact that patients were measured while taking regular medication. We can also not be sure of the long-term persistence of the improvements we observed [45]. Concerns about this have been raised in the past [44]. Nevertheless, our data suggest that the results are unlikely to be simply an effect of improvement in freezing of gait (FoG), as subjects with FoG were a minority in our sample. Similarly, it remains unlikely that the improvement in gait within the 85 Hz condition was simply due to fewer side effects. Although very few side effects were observed in this condition (see Table 2), gait speed in the 85 Hz condition surpassed gait speed in the patient’s default setting, which had the lowest number of side effects and a frequency above 85 Hz for all but one subject. With regard to pulse width we have to acknowledge that the 90 µs setting was not tolerated by many patients, thus our findings here are of limited generalizability. We also did not perform a formal testing of a 60µs pulse width while this turned out to be the value most frequently used by patients in their regular stimulator setting (cf. supplementary Table 1).

DBS with its short latency seems predisposed for regular and objective assessment methods and one may advocate for collecting more information on gait in iPD patients in general. Objective gait measuring approaches like the one applied by us could pave the way to reduce outpatient visits and healthcare costs [46] and may make laborious and sometimes unreliable patient diaries [47, 48] obsolete. They may also improve the assessment of benefits obtained from occupational or physical therapy. The list of possible features that sensors can measure is extensive [49] and feature combinations and machine learning techniques^39^ may even improve utility. Sensor recordings could also empower patients for informed decision making when combined with feedback from healthcare professionals, so that more individual therapy plans can be developed. DBS settings could even be adjusted by patients themselves based on such measurements.

## Conclusion

Using measurements from mobile gait sensors we show that gait speed, stride length and leg lift can be improved when stimulation frequency is lowered below the current standard value of 130 Hz to 85 Hz. Higher stimulation pulse widths can have a beneficial effect on leg lift when tolerated by patients. We aim to provide clinicians with evidence-based strategies that can be translated to clinical practice for effectively managing iPD patients’ symptoms and improving their overall well-being.

## Electronic supplementary material

Below is the link to the electronic supplementary material.

Supplementary Material 1

Supplementary Material 2

## Data Availability

All analyses and results are available from an online repository (https://github.com/dpedrosac/DBSgait). The data that support the findings of this study are not openly available due to reasons of sensitivity and are available from the corresponding author upon reasonable request.

## Abbreviations

DBS: Deep brain stimulation
FoG: Freezing of gait
iPD: Idiopathic parkinsons disease
LFP: Local field potential
MDS-UPDRS: Movement disorder society sponsored revision of the unified parkinson’s disease rating scale
STN: Nucleus subthalamicus
TUG: Timed up and go test
