# Supplementary material for: Subthalamic 85 Hz deep brain stimulation improves walking pace and stride length in Parkinson’s disease patients
Source: Neurol Res Pract. 2023 Aug 10;5:33. doi: 10.1186/s42466-023-00263-7 (PMC10413698; doi:10.1186/s42466-023-00263-7)
Supplement: Supplementary file 1 — Supplementary Material 1 [file 42466_2023_263_MOESM1_ESM.docx]

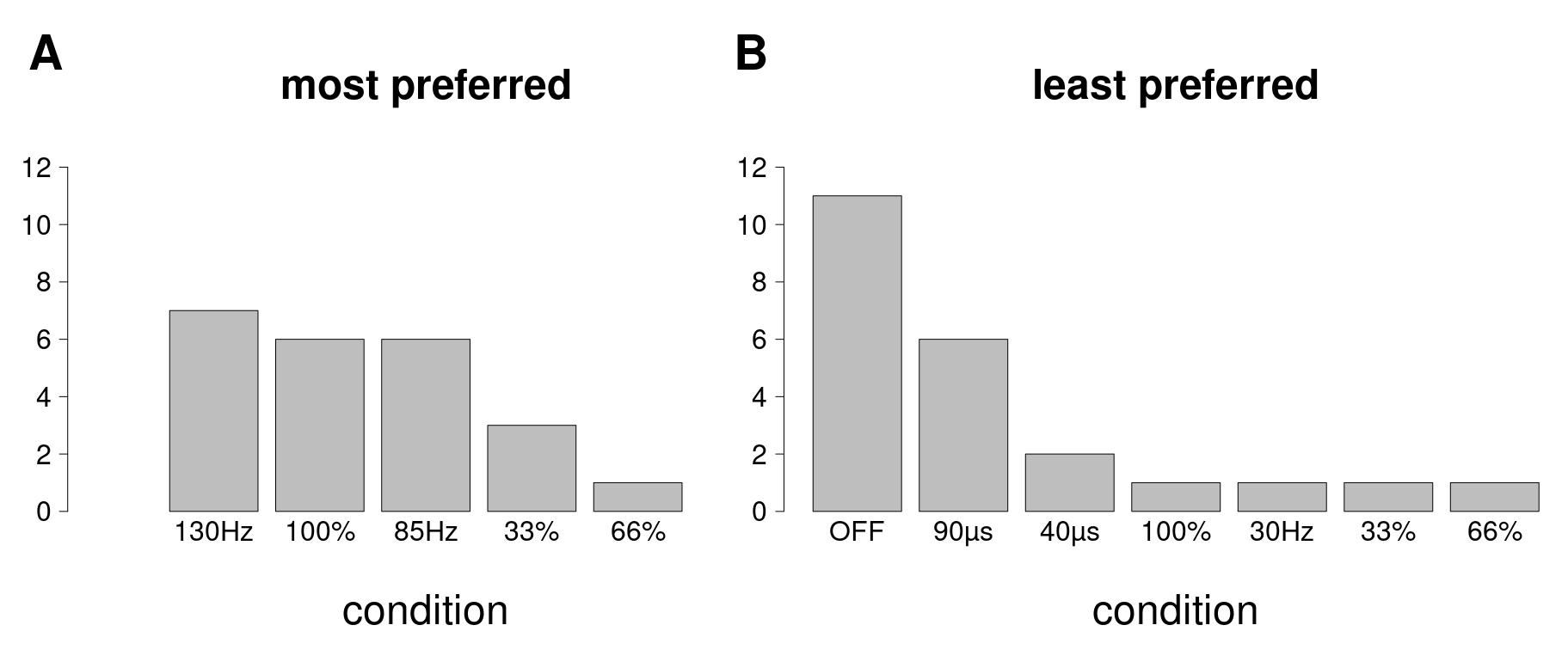


Supplementary figure 1: Histograms (absolute counts) of most and least preferred DBS conditions.


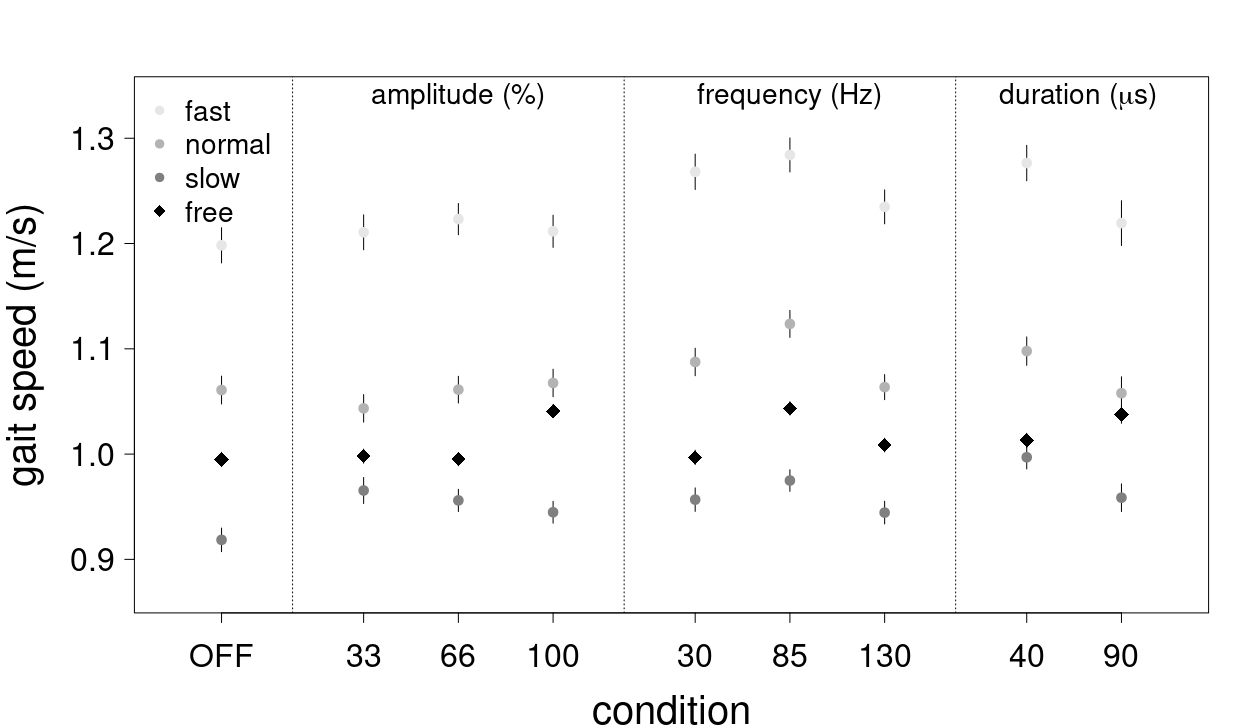


Supplementary figure 2: Gait speed estimates in the individual gait tasks (slow, fast, normal and free gait). Depicted are all measured stimulation conditions. OFF is no DBS stimulation, 33, 66 and 100 refer to the percentage in amplitude of the original stimulation settings of the patient. 30, 85 and 130 denote the stimulation frequency (Hz) whereas 40 and 90 refer to the stimulation pulse widths (µs). Error bars are two standard errors of the estimated difference between each condition and the reference condition (OFF). The error for the reference condition is estimated from the average of all other errors.
