# Supplementary material for: Subthalamic 85 Hz deep brain stimulation improves walking pace and stride length in Parkinson’s disease patients
Source: Neurol Res Pract. 2023 Aug 10;5:33. doi: 10.1186/s42466-023-00263-7 (PMC10413698; doi:10.1186/s42466-023-00263-7)
Supplement: Supplementary file 2 — Supplementary Material 2 [file 42466_2023_263_MOESM2_ESM.docx]

*Supplementary Table 1: Clinical data of every patient along with preset stimulation settings.*

| **ID** | **1** | **2** | **3** | **4** | **5** | **6** | **7** | **8** | **9** | **10** | **11** | **12** | **13** | **14** | **15** | **16** | **17** | **18** | **19** | **20** | **21** | **22** | **23** |
| --- | --- | --- | --- | --- | --- | --- | --- | --- | --- | --- | --- | --- | --- | --- | --- | --- | --- | --- | --- | --- | --- | --- | --- |
| **age** | 65 | 58 | 62 | 57 | 52 | 55 | 72 | 56 | 56 | 43 | 60 | 56 | 52 | 46 | 63 | 58 | 57 | 56 | 56 | 64 | 68 | 41 | 58 |
| **sex** | m | w | m | m | m | m | w | m | m | w | m | m | m | w | w | w | w | m | w | w | m | m | w |
| **height** | 191 | 166 | 183 | 181 | 182 | 176 | 158 | 178 | 182 | 164 | 178 | 172 | 182 | 163 | 164 | 172 | 167 | 202 | 170 | 173 | 186 | 167 | 166 |
| **disease duration (years)** | 18 | 20 | 11 | 19 | 10 | 8 | 8 | 9 | 7 | 11 | 10 | 6 | 10 | 5 | 25 | 11 | 10 | 6 | 3 | 16 | 12 | 7 | 6 |
| **UPDRS part III ON** | 14 | 13 | 15 | 10 | 38 | 22 | 25 | 9 | 9 | 14 | 4 | 10 | 13 | 4 | 7 | 4 | 25 | 35 | 23 | 9 | 19 | 7 | NA |
| **UPDRS part III OFF** | 31 | 22 | 43 | 13 | 56 | 48 | 39 | 13 | 17 | 27 | 15 | 31 | 21 | 29 | 25 | 8 | 49 | 52 | 52 | 17 | 47 | 23 | NA |
| **more affected side** | R | R | R | L | R | R | L | R | R | L | L | R | R | R | R | R | L | L | L | L | R | L | R |
| **type** | BR | BR | EQ | BR | EQ | BR | TD | BR | BR | EQ | EQ | EQ | BR | BR | TD | BR | BR | BR | BR | BR | EQ | BR | BR |
| **FOG** | yes | no | no | no | yes | yes | no | no | no | no | no | no | no | no | no | no | no | no | no | no | yes | no | no |
| **amplitude left (mA)** | 4.0 | 1.2 | 2.0 | 2.7 | 3.6 | 2.6 | 4.2 | 1.7 | 1.3 | 1.5 | 2.6 | 2.8 | 3.5 | 3.8 | 3.0 | 2.6 | 0.9 | 2.7 | 2.6 | 3.4 | 2.4 | 2.5 | 4.6 |
| **amplitude right (mA)** | 3.5 | 1.2 | 2.0 | 2.8 | 1.3 | 1.9 | 3.4 | 1.7 | 1.3 | 1.7 | 3.4 | 1.7 | 1.3 | 2.0 | 2.8 | 2.6 | 0.6 | 4.0 | 3.0 | 2.8 | 2.1 | 3.1 | 2.6 |
| **frequency left (Hz)** | 100 | 130 | 130 | 120 | 132 | 130 | 185 | 130 | 130 | 130 | 130 | 130 | 130 | 130 | 130 | 130 | 130 | 130 | 130 | 130 | 130 | 79 | 130 |
| **frequency right (Hz)** | 100 | 130 | 130 | 120 | 159 | 130 | 185 | 130 | 130 | 130 | 130 | 130 | 130 | 130 | 130 | 130 | 130 | 130 | 130 | 130 | 130 | 79 | 130 |
| **pulse width (µs)** | 60 | 60 | 60 | 60 | 30 | 60 | 50 | 60 | 60 | 60 | 60 | 60 | 60 | 60 | 60 | 60 | 60 | 60 | 60 | 60 | 60 | 50 | 40 |

Abbrevations: ID – identity; *FOG – freezing of gait; UPDRS – Unified Parkinson’s Disease Rating Scale; pt. – part; mA – milliampere; Hz – Hertz; µs – Microseconds; m – male; f- female; BR – bradykinetic-rigid; TD – tremor dominant; EQ – equivalence type; L – left; R – right*

*Supplementary Table 2: Stimulation parameters tested in the experiment*

| **Amplitude** | **Frequency** | **Pulse width** |
| --- | --- | --- |
| 100 %* | 130 Hz | 90 µs |
| 66 %* | 85 Hz | 40 µs |
| 33 %* | 30 Hz |  |
| OFF |  |  |

*refers to the originally programmed stimulation intensity of each patients’ stimulator.

*Supplementary Table 3:*

*Number of patients who did not tolerate a particular DBS setting. All initially included patients (n=27) are considered.*

|  | **Amplitude (%)** | | | | **Frequency (Hz)** | | | **Pulse width (µs)** | |
| --- | --- | --- | --- | --- | --- | --- | --- | --- | --- |
|  | **OFF** | **33** | **66** | **100** | **30** | **85** | **130** | **40** | **90** |
| **number of patients not tolerating the condition** | 4 | 5 | 2 | 1 | 6 | 2 | 3 | 4 | 14 |
